# OH-defects in multiple-doped orthoenstatite at 4–8 GPa: filling the gap between pure and natural systems

**DOI:** 10.1007/s00410-015-1133-8

**Published:** 2015-04-04

**Authors:** Roland Stalder, Asiya Karimova, Jürgen Konzett

**Affiliations:** Institut für Mineralogie und Petrographie, Universität Innsbruck, Innrain 52 f, 6020 Innsbruck, Austria

**Keywords:** Enstatite, Geobarometer, High pressure, Water incorporation, OH-defects

## Abstract

OH-defects in orthoenstatite were studied experimentally between 4 and 8 GPa at 1150 °C in the system CaO–MgO–Al_2_O_3_–SiO_2_–Cr_2_O_3_–Na_2_O, leading to phase assemblages enstatite ± forsterite ± diopside ± garnet. In enstatite coexisting with garnet, total OH is negatively correlated with pressure. Conversely, in Al-poor systems without garnet, total OH is positively correlated with pressure, and both trends intersect around 8 GPa and ~1000 wt ppm H_2_O. IR-spectra of enstatite reveal several pressure sensitive features, such as (1) the absorbance of the absorption band at 3687 cm^−1^, (2) the band position near 3400 cm^−1^ and (3) the ratio (*A*_3240–3570_/*A*_3240–3730_) and their application as geobarometer in natural samples are evaluated. For garnet-bearing phase assemblages, the band ratio (*A*_3240–3570_/*A*_3240–3730_) in orthoenstatite defines a pressure trend in between that observed in the pure system MgO–SiO_2_–H_2_O and that found in orthopyroxenes from natural mantle peridotites, suggesting that the application of IR-spectra as proxy for pressure is justified.

## Introduction

Hydrous defects make nominally anhydrous minerals (NAMs) important hosts for water in the Earth’s mantle and affect their physical properties such as rheology (Mackwell et al. 1985; Hirth and Kohlstedt 1996; Mei and Kohlstedt 2000) and electrical conductivity (Karato 1990; Wang et al. 2006; Schlechter et al. 2012). Although the nature of OH-defects is a complex function of many fundamental petrological parameters such as pressure, temperature, silica activity, oxygen fugacity (e.g. Withers and Hirschmann 2008), water activity and phase assemblage, certain features of the OH-defect incorporation may be used to decipher the physical conditions of NAM-formation/equilibration. Hydrous defect formation in chemically pure systems has been studied extensively during the past decades and is comparably well understood, but often not directly applicable to nature due to the absence of components that are critical for defect formation. In contrast, defect formation in natural systems is not as well understood as a result of the chemical complexity of these systems.

Here, we present results from an experimental study at 4–8 GPa in the system CaO–MgO–Al_2_O_3_–SiO_2_–Cr_2_O_3_–Na_2_O, which is intermediate in its complexity between the strongly simplified systems such as MgO–SiO_2_–H_2_O and the natural peridotitic systems. In this system, orthoenstatite ± diopside ± forsterite ± garnet coexist, reproducing the assemblage typically stable in peridotitic upper mantle at depths <~250 km. The major aim of this study is to investigate the OH-defect inventory of orthopyroxene as major host of OH-defects in upper mantle minerals as a function of pressure and bulk composition. Orthopyroxene has a high modal proportion in average peridotites of the uppermost mantle (olivine > orthopyroxene > clinopyroxene > Al-phase; Maaløe and Aoki 1977; Frey and Prinz 1978; Oehm et al. 1983) and high OH-contents (OH_cpx_ > OH_opx_ ≫ OH_ol_ ≈ OH_Al-phase_, e.g. Ingrin and Skogby 2000; Peslier et al. 2002; Peslier and Luhr 2006; Grant et al. 2007). Thus, in the majority of mantle peridotites up to ~200 km depth, orthopyroxene is the most important OH-carrier, if nominally hydrous phases are not present.

In this study, OH-defects are characterised and quantified by FTIR spectroscopy. OH-dipoles revealed by IR absorptions are produced by point defects, where protons act as charge compensation for metal vacancies or impurities. Since oxygen is the only charge balancing anion in the crystal, the relevant component for petrology and geochemistry is water. Therefore, throughout this article, the terms “OH-incorporation”, “hydrogen incorporation”, “proton incorporation” and “water incorporation” are used synonymously. Concentrations are given as wt ppm water.

## Experimental procedure

For the starting materials, high-purity oxides and carbonates were mixed in appropriate stoichiometric proportions (Table 1) and these mixtures were decarbonated by step-wise firing to 1000 °C. Approximately 20 mg solid starting material was then loaded into a platinum capsule with an outer (inner) diameter of 3.0 (2.6) mm, respectively. Prior to sealing water was added with a micro-syringe in order to yield a silicate-to-water ratio of 85:15.

**Table 1:** Run conditions, anhydrous starting mixtures and phase assemblages

Run	Pressure (GPa)	SiO_2_	MgO	CaO	Al_2_O_3_	Cr_2_O_3_	Na_2_O	Phase assemblage
AK036	6	49.45	43.38	4.75	1.68	0.39	0.33	En + Di
AK037	6	49.45	43.38	4.75	1.68	0.39	0.33	En + Di
AK038	8	49.45	43.38	4.75	1.68	0.39	0.33	En + Di
AK040	4	49.56	41.92	6.25	1.59	0.36	0.31	En + Di
AK043	8	49.34	44.33	3.75	1.80	0.42	0.35	En + Di
AK044	4	49.34	44.33	3.75	1.80	0.42	0.35	En
AK052	4	44.15	47.96	2.36	5.28	0.19	0.07	En + Fo
AK056	8	43.31	45.17	2.18	7.14	0.56	1.63	En + Fo + Gt
AK057	6	43.31	45.17	2.18	7.14	0.56	1.63	En + Fo + Gt
AK059	8	44.58	49.71	2.40	2.03	0.30	0.97	En + Fo + Gt^a^
AK060	4	43.33	45.16	2.18	7.14	0.56	1.63	En + Fo + Gt
AK064	4	40.56	42.41	2.82	12.11	1.06	1.03	En + Fo + Gt^a^

*En* enstatite, *Di* diopside, *Fo* forsterite, *Gt* garnet

^a^Gt was not observed, but inferred from bulk and crystal chemistry considerations. All experimental charges contained a solid–water mixture in an approximate weight ratio of 85:15

High-pressure/high-temperature experiments were performed with a 1000-ton Walker-type multianvil device at the University of Innsbruck using 25/15 assemblies with MgO–Cr_2_O_3_ octahedra and graphite furnaces. Experimental and calibration conditions are similar to those described by Rubie et al. (1993) and Keppler and Frost (2005), and the pressure is estimated to be correct within 0.2 GPa. Temperature was measured with a Pt_100_–Pt_90_Rh_10_ thermocouple, and both pressure and temperature were computer-controlled during the entire duration of the runs. For each run, the temperature was initially raised to 1300 °C and held for 3 h followed by a decrease to 1150 °C at a rate of 10 °C/h. As soon as the final temperature was reached, runs were terminated by shutting off the heating power. After removal from the assembly, the capsules were weighed, pierced, dried and weighed again to determine the amount of fluid present during the experiment and to check for any fluid loss. This test showed that with the exception of run AK38 (Table 1), no significant fluid loss had occurred. Run AK38 still produced large and euhedral orthopyroxene grains which was taken as indication for the presence of fluid during the experiments.

Run products were inspected under an optical microscope and analysed without further preparation or grinding by X-ray diffraction using a Bruker-AXS D8 powder diffractometer equipped with an energy dispersive SOL-X detector in parallel beam optics mode, or a Siemens D5000 powder diffractometer with a scintillation counter in Bragg–Brentano geometry in the 2*θ* range between 2° and 70° (Cu-K_α_ radiation, generator settings: 40 kV/40 mA, step size: 0.01° 2*θ*). Single crystals of enstatite were handpicked under a binocular microscope and oriented in a thermoplastic resin parallel (010) and (100). The proper alignment was checked using a polarisation microscope with conoscopic illumination and a posteriori verified by IR-spectroscopy based on the lattice overtones (see analytical section). All crystal sections were polished on both sides, reaching a final thickness of 50–300 µm. After preparation, the thermoplastic resin was removed by rinsing in acetone. Crystals of accompanying phases generally were too small to get aligned and analysed by FTIR, because the study was focussed on OH-defects in enstatite, and therefore, the bulk composition was adjusted to produce a large proportion (and hence large crystals) of enstatite.

## Analytical procedure

### Electron microprobe

To determine the composition of the run products, hand-picked crystals of sufficient size were embedded in epoxy resin and analysed with a JEOL JXA-8100 electron microprobe in the wavelength-dispersive analytical mode. Analytical conditions were 15 kV acceleration voltage and 10 nA beam current with measurement durations of 20 and 10 s on peaks and background, respectively, for each X-ray line. The following standards were used: synthetic quartz (Si), synthetic corundum (Al), natural diopside (Mg, Ca), jadeite (Na) and chromite (Cr). Detection limits were around 100 ppm (wt) for Si, Al and Ca, 130 ppm (wt) for Mg and Na and 200 ppm for Cr. Inspection using BSE imaging and analyses of traverses across individual orthopyroxene crystals revealed no significant compositional zoning. Analytical results are given in Table 2.

**Table 2:** Chemical compositions (wt%) of crystalline phases determined by electron microprobe (analytical error in parentheses)

Phase	Run	analyses	SiO_2_	MgO	CaO	A1_2_O_3_	Cr_2_O_3_	Na_2_O	Mole Al/(Al + Cr)	Total wt%
En	AK036	17	59.77 (0.24)	38.18 (0.17)	0.83 (0.12)	0.33 (0.06)	0.29 (0.05)	0.04 (0.01)	0.63	99.43
En	AK037	22	59.72 (0.26)	38.18 (0.23)	0.97 (0.13)	0.29 (0.10)	0.26 (0.05)	0.03 (0.01)	0.62	99.45
En	AK038	4	59.82 (0.23)	38.21 (0.28)	0.91 (0.03)	0.39 (0.02)	0.25 (0.06)	0.02 (0.01)	0.70	99.59
En	AK040	13	59.72 (0.18)	38.18 (0.25)	0.92 (0.18)	0.15 (0.04)	0.34 (0.12)	0.02 (0.01)	0.40	99.32
En	AK043	15	59.35 (0.25)	40.22 (0.17)	0.77 (0.08)	0.40 (0.09)	0.26 (0.08)	0.03 (0.02)	0.70	101.04
En	AK044	16	59.77 (0.34)	40.59 (0.27)	0.32 (0.04)	0.14 (0.05)	0.26 (0.09)	0.01 (0.01)	0.45	101.08
En	AK052	9	59.00 (0.43)	40.70 (0.04)	0.28 (0.04)	0.70 (0.21)	0.32 (0.08)	0.03 (0.01)	0.77	100.79
En	AK056	16	58.97 (0.28)	40.69 (0.27)	0.36 (0.11)	0.50 (0.11)	0.16 (0.07)	0.11 (0.03)	0.82	100.80
En	AK057	5	58.78 (0.36)	39.95 (0.50)	0.34 (0.01)	0.79 (0.07)	0.21 (0.03)	0.06 (0.01)	0.85	100.12
En	AK059	5	58.83 (0.25)	39.78 (0.39)	0.25 (0.02)	0.43 (0.05)	0.25 (0.01)	0.07 (0.01)	0.72	99.60
En	AK060	10	57.45 (0.47)	38.63 (0.41)	0.23 (0.01)	2.21 (0.41)	1.35 (0.21)	0.07 (0.02)	0.71	99.95
En	AK064	7	55.94 (0.38)	38.18 (0.43)	0.20 (0.01)	4.46 (0.39)	1.02 (0.16)	0.04 (0.02)	0.87	99.86
Di	AK036	2	59.79 (0.52)	18.45 (0.42)	20.63 (0.17)	3.92 (0.59)	0.37 (0.02)	0.72 (0.11)	0.94	99.87
Di	AK038	6	56.56 (0.20)	21.22 (0.36)	21.45 (0.41)	0.56 (0.04)	0.50 (0.04)	0.28 (0.05)	0.62	100.58
Di	AK040	2	55.82 (0.62)	22.12 (0.21)	20.27 (0.61)	1.55 (0.13)	0.23 (0.08)	0.12 (0.02)	0.91	100.10
Di	AK043	15	55.59 (0.33)	21.23 (0.32)	22.11 (0.29)	0.66 (0.08)	0.60 (0.14)	0.36 (0.05)	0.62	100.55
Gt	AK057	3	43.18 (0.77)	26.53 (0.32)	5.83 (0.23)	23.33 (0.18)	1.15 (0.19)	0.03 (0.02)	0.97	100.05

*En* enstatite, *Di* diopside, *Gt* garnet

### FTIR spectroscopy and trace water quantification

Mid-infrared absorption spectra were recorded at room temperature in transmission mode using a Bruker Vertex 70 FTIR spectrometer, coupled to a Hyperion 3000 microscope equipped with liquid nitrogen-cooled MCT-D316-025 (mercury cadmium telluride) detector, a silicon carbide (SiC) global source, a KBr beam splitter and a ZnSe wire grid polarizer. Each spectrum was acquired by 32 scans in the range between 550 and 7500 cm^−1^ with a spectral resolution of 2 cm^−1^. Spectra were recorded over a large portion of each crystal as average values, representing a volume comparable to that analysed by electron microprobe. As the chemical zonation with respect to metal impurities was rather small, no attempt to quantify the zonation in OH-content was made. Special attention was drawn to the spectral region of OH-stretching vibrations (3000–3700 cm^−1^) and the lattice overtones (1200–2200 cm^−1^), the latter being used to confirm the polarisation direction by comparison with previously published spectra (Prechtel and Stalder 2012; Mosenfelder and Rossman 2013) and to independently check the thickness of the crystal section by the intensity of the overtones. OH-defect contents, expressed as wt ppm water, were calculated using the calibrations of Bell et al. (1995), Libowitzky and Rossman (1997) and Stalder et al. (2012). Results are summarised in Table 3.

**Table 3:** IR integral absorbances (*nα* + *nβ* + *nγ*) normalised to 1 mm thickness and calculated water contents

Run	Pressure (GPa)	*A*_3570–3730_ (cm^−2^)	*A*_3240–3570_ (cm^−2^)	*A*_3240–3730_ (cm^−2^)	$\frac{{A_{{3240\text{-}3570}} }}{{A_{{3240\text{-}3730}} }}$	wt ppm H_2_O		
						LR97	B95	Stl2
AK036	6	20.0	351.6	371.6	0.946	712	845	1122
AK037	6	27.2	260.6	287.8	0.906	590	646	952
AK038	8	36.0	366.3	402.3	0.911	761	810	1232
AK040	4	13.7	236.7	250.4	0.945	484	576	761
AK043	8	67.7	775.5	843.2	0.920	1591	1736	2543
AK044	4	20.4	253.5	273.9	0.926	550	629	881
AK052	4	50.2	350.0	400.2	0.875	814	831	1319
AK056	8	90.6	398.9	489.5	0.815	1132	1067	1935
AK057	6	105.0	539.1	644.1	0.837	1380	1286	2289
AK059	8	100.5	418.3	518.8	0.806	1204	1073	2077
AK060^a^	4	(68.8)	(430.7)	(499.5)	(0.862)	1523	1456	2460
AK064	4	128.8	682.4	811.2	0.841	1586	1524	2554

LR97 = Libowitzky and Rossman (1997), B95 = Bell et al. (1995), Stl2 = Stalder et al. (2012)

^a^Integral absorbances *nα* + *nγ* (*nβ* was not measured). For quantification of the water content, *nβ* was assumed to be similar to AK064

## Results

### Run products

All runs produced orthoenstatite with crystals up to several 100 µm in size, free fluid and amorphous quench material. Depending upon the composition of the starting material and the pressure, diopside, forsterite and/or garnet appeared in addition (Table 1). In some runs with high Al bulk compositions (e.g. AK52, AK60, AK64), X-ray diffraction analysis indicated the presence of sheet silicates with 12 Å spacing. These sheet silicates, however, could not be detected by optical inspection and, hence, are thought to be metastable quench products. As the detection of accompanying phases was difficult if their modal proportion was very low, additional information based on crystal chemistry of the observed phases was further considered. Ca-saturation of enstatite based on the diopside–enstatite solvus (Nickel and Brey 1984) was taken as indication for the presence of diopside in cases when this phase could not be detected due to its low modal proportions. This issue will be discussed in more detail in the next section. Likewise, the Al-content of enstatite served as sensitive indicator for the presence or absence of garnet (cf. Lane and Ganguly 1980). In runs where two pyroxenes coexist, a negative correlation of Al/(Cr + Al) in clinopyroxene with pressure was observed (Table 2), in agreement with results obtained by Nimis and Taylor (2000).

### IR-spectra of orthoenstatite

**Fig. 1:**
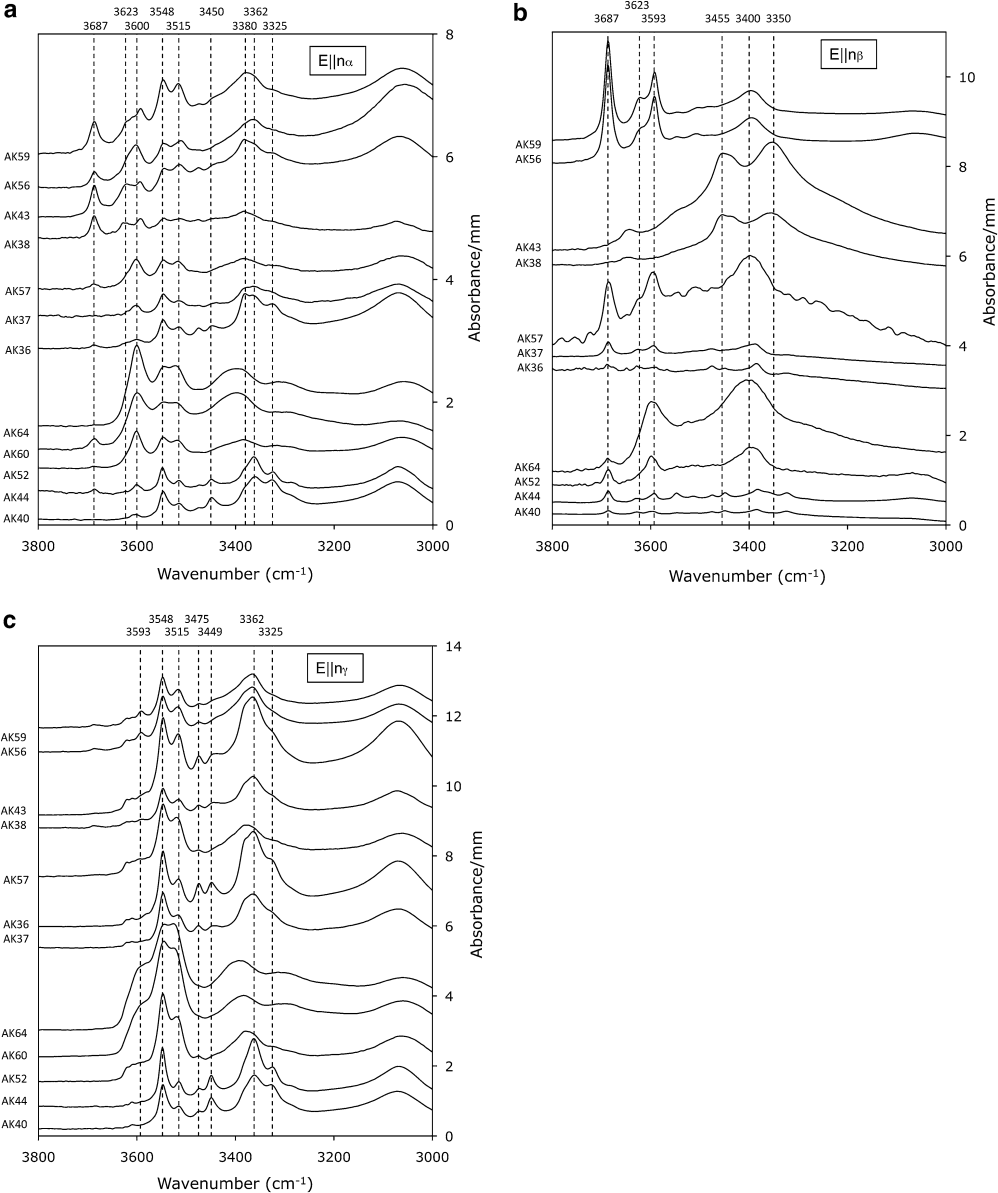
Polarised IR-spectra of OH-stretching vibrations for *E*||*nα* (**a**), *E*||*nβ* (**b**) and *E*||*nγ* (**c**) recorded on oriented enstatite single crystal sections. Most prominent bands are highlighted by *broken lines*. Spectra are not baseline corrected, normalised to 1 mm thickness, grouped according to pressure (details see Tables 1, 2) and offset for clarity

**Fig. 2:**
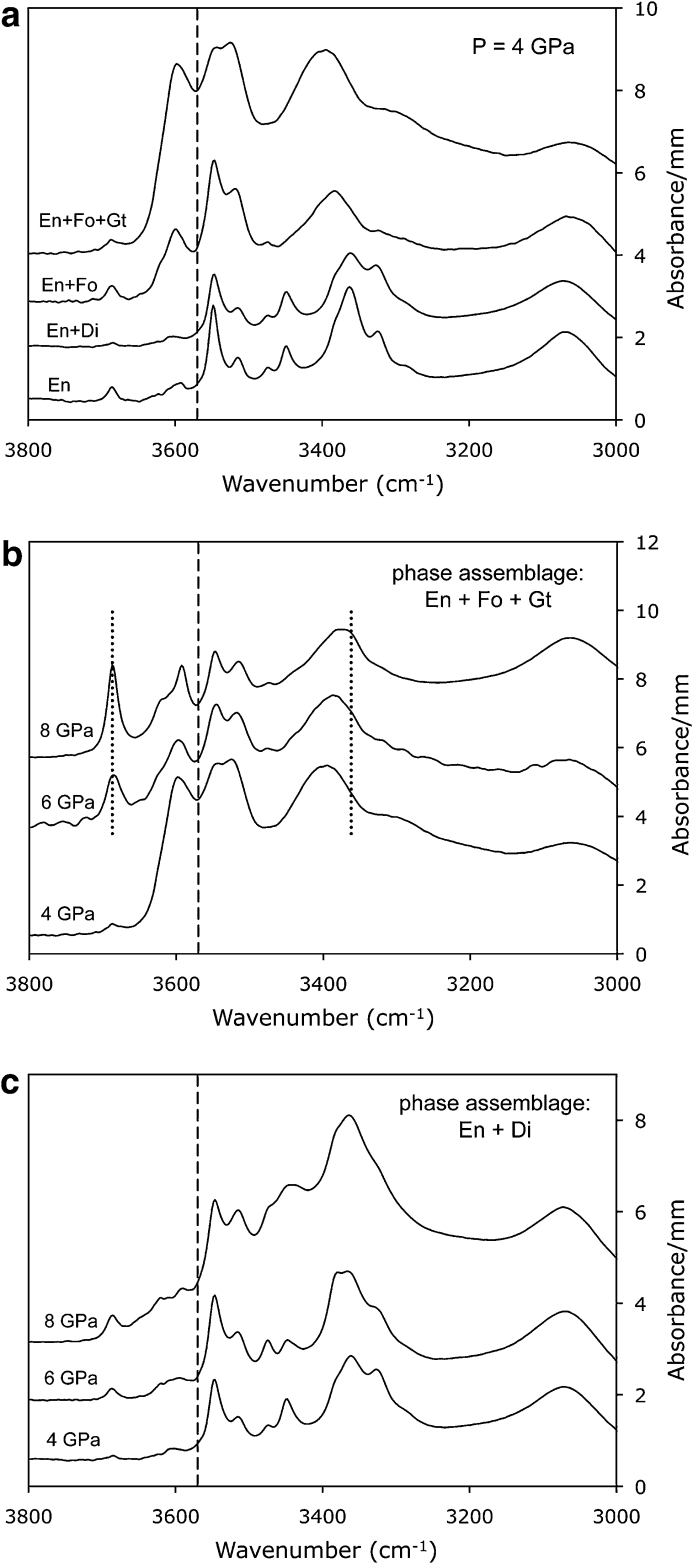
IR-spectra of OH-stretching vibrations shown as *E*||*nα* + *E*||*nβ* + *E*||*nγ* from polarised measurements on oriented crystal sections. Comparison of different phase assemblages at constant pressure (**a**), pressure dependence of the phase assemblage En + Fo + Gt (**b**) and En + Di (**c**). When data from two runs with the same conditions were available (e.g. AK36/37, AK38/43 and AK56/59), average spectra are shown. Bands assigned to OH associated with metal vacancies in pure enstatite are shown in **b** as *dotted lines*. The subsequently used division between high-wavenumber and low-wavenumber bands at 3570 cm^−1^ is also displayed as *broken line*. Spectra are normalised to 1 mm thickness and offset for clarity

**Fig. 3:**
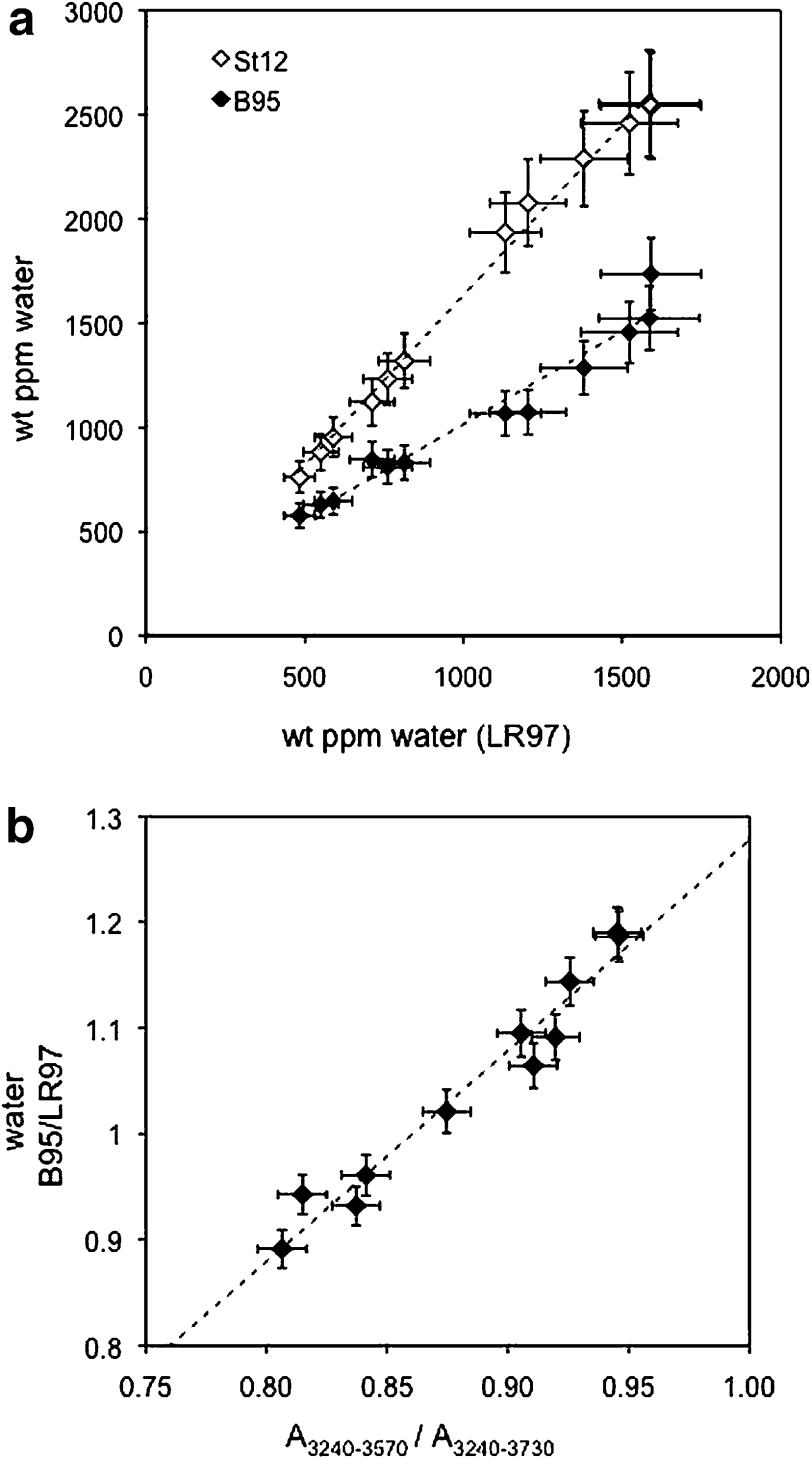
Water contents of enstatite using the calibrations of Bell et al. (1995) = B95 and Stalder et al. (2012) = St12 plotted against water contents using the calibration of Libowitzky and Rossman (1997) = LR97 (**a**). A higher proportion of low-wavenumber bands (*A*_3240–3570_) results in an overestimation of B95 compared to LR97 (**b**)

IR-spectra of orthoenstatite exhibit many absorption bands between 3000 and 3700 cm^−1^ (Fig. 1), some of them amalgamating to broad absorption features similar to those known from natural specimens (e.g. Bell et al. 1995; Prechtel and Stalder 2012). Band positions for *E*||*nα* are more similar to *E*||*nγ* than to *E*||*nβ* (in accord with Prechtel and Stalder 2011, 2012), but considerable deviations from this behaviour are observed for samples with high Al-content. High-wavenumber bands >3570 cm^−1^ are most strongly developed for *E*||*nβ*, but in some samples also show significant contributions for *E*||*nα*. IR-spectra summed over all three polarisation directions *E*||*nα* + *E*||*nβ* + *E*||*nγ* show systematic changes dependent upon phase assemblage (Fig. 2a) and pressure (Fig. 2b, c). Specifically, high-wavenumber absorption bands such as the one at 3687 cm^−1^ tend to increase with pressure in comparison with low-wavenumber absorption bands, and the band near 3600 cm^−1^ shows a positive correlation between intensity and bulk Al-content, culminating in maximum intensities in garnet-bearing assemblages (Fig. 2a). Integral absorbances for bands >3570 cm^−1^ and <3570 cm^−1^ are listed in Table 3, and water concentrations determined by the calibrations of Bell et al. (1995), Libowitzky and Rossman (1997) and Stalder et al. (2012) are plotted in Fig. 3. With the calibration of Bell et al. (1995), spectra with a larger proportion of low-wavenumber bands yield systematically higher water contents than with the calibration of Libowitzky and Rossman (1997) (Fig. 3b), which is in agreement with Mosenfelder and Rossman (2013). In general, water concentrations of samples synthesised under similar *P*–*T*–*x* conditions are in good agreement. The only exception is the discrepancy between AK38 and AK43 (En + Di at 8 GPa), which was caused by partial fluid loss from AK38.

## Discussion

### Di–En solvus

**Fig. 4:**
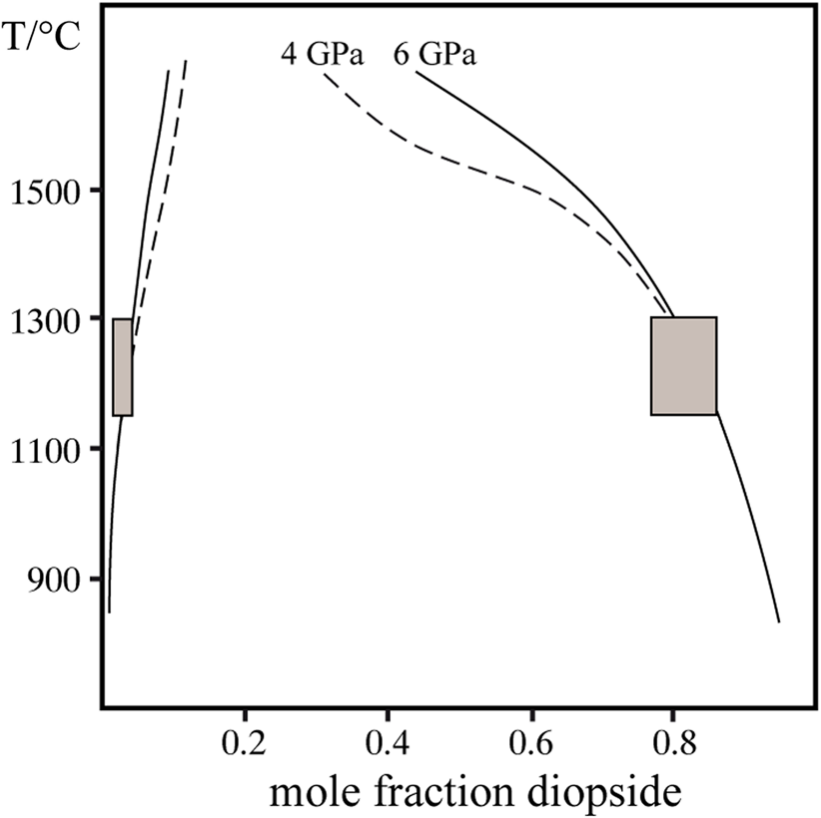
Range of diopside–enstatite solid solution (*grey boxes*) in runs, where both pyroxenes coexist. Solvus for 4 GPa (*broken line*) and 6 GPa (*solid line*) after Nickel and Brey (1984) for comparison

In charges where two pyroxenes coexist, enstatite (En) contains on average 3 mol% diopside (Di) component and diopside contains between 15 and 22 mol% enstatite component (Table 2; Fig. 4). The latter value is slightly higher than expected for a temperature of 1150 °C in the system CaO–MgO–SiO_2_ (Nickel and Brey 1984). This discrepancy, however, can be explained if the temperature history of the experiments is considered: the observed range in pyroxene Ca-contents is thought to be a result of continuous pyroxene crystallization over a temperature interval of 150 °C (i.e. from 1300 to 1150 °C, cf. Figure 4) and reflects incomplete re-equilibration of Ca in orthopyroxene at the final run temperature of 1150 °C. This assumption is consistent with temperatures of 1275 °C derived from two-pyroxene thermometry based on the calibration by Nimis and Taylor (2000). An excess diopside component in enstatite incorporated at an early stage of the experiments, however, is not likely to affect water incorporation into enstatite to any significant degree as will be discussed in the next paragraph.

### Water in enstatite

**Fig. 5:**
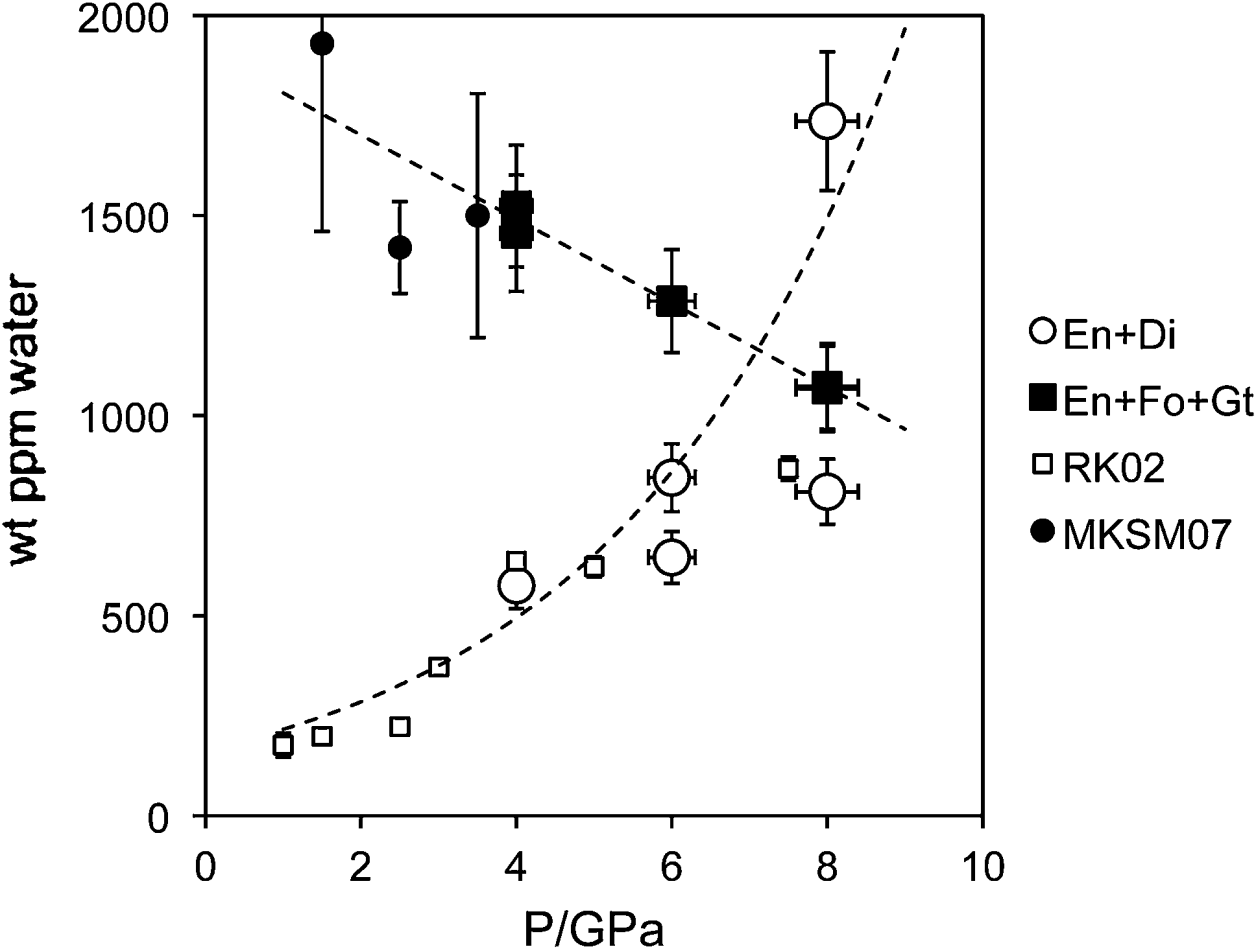
Water content (as OH-defects) in enstatite against pressure using the calibration of Bell et al. (1995). RK02 and MKSM07 are data for pure and Al-saturated orthoenstatite at 1100 °C from Rauch and Keppler (2002) and Mierdel et al. (2007), respectively. The large discrepancy between the two 8 GPa runs with (En + Di) are probably caused by partial fluid loss from AK38

Water concentrations show a positive correlation with pressure for the phase assemblage enstatite + diopside (Fig. 5), which is in good agreement with data for pure orthoenstatite obtained by Rauch and Keppler (2002). Therefore, it is concluded that the diopside component in enstatite has no significant influence on OH-incorporation, at least at small molar fractions of diopside as in the present study (En_97_Di_3_ in run AK40 and En_99_Di_1_ in AK44, both showing water concentration identical within the analytical error, see Table 3). The diopside component itself has generally been shown to be a poor water carrier compared to the enstatite component, because (1) pure diopside shows rather low OH-defect contents unless doped with mono- or trivalent metal cations (Stalder and Ludwig 2007; Purwin et al. 2009), (2) in the undoped CaO–MgO–SiO_2_ system, the water partition coefficient between enstatite and diopside is >1, and (3) diopside in the phase assemblage Di + En has a higher concentration of hydrous defects than in the assemblage Di + Wo, where diopside is close to stoichiometry (Karimova and Stalder 2013), which means that a dissolved enstatite component in diopside has a stronger influence on OH in diopside than a dissolved diopside component in enstatite.

**Fig. 6:**
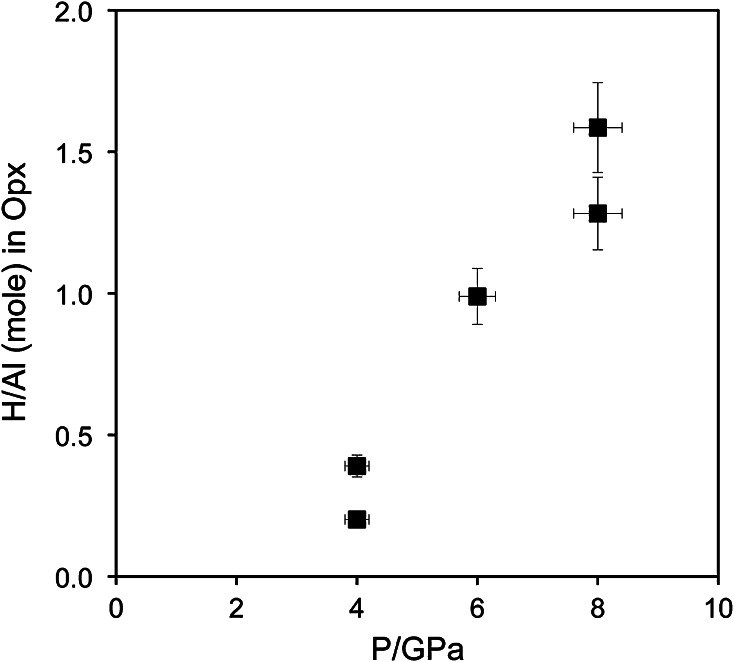
Role of Al for incorporation of H in enstatite in the phase assemblage En + Fo + Gt as function of pressure. H-concentration was calculated from the water content using the calibration of Bell et al. (1995)

Water incorporation into enstatite also depends on the coexisting phase assemblage, but this trend is governed by the Al-content in enstatite rather than the coexistence with an Al-rich phase. Enstatite with an Al-content just below Al-saturation incorporates similar amounts of OH-defect contents as at Al-saturation, and the high water contents in enstatite coexisting with garnet are due to the high Al-contents in enstatite compared to Al-free (or Al-poor) systems. The observed negative correlation with pressure is opposite to the trend observed for enstatite coexisting with diopside only. Both, Al-saturated and undersaturated trends intersect near 8 GPa and approximately 1000 wt ppm H_2_O (Fig. 5). Taken into account that Al-incorporation in enstatite is negatively correlated with pressure in the garnet stability field (Lane and Ganguly 1980), this means that the H^+^/Al^3+^ ratio increases with pressure (Fig. 6). This trend can, however, not be interpreted as a more effective coupling of H^+^ to Al^3+^ as the increased incorporation of protons also is observed in the Al-free system (Rauch and Keppler 2002), and the reduced Al^3+^-content can be related to reduced Tschermaks substitution at high pressure. In particular, at 4 GPa, most of the Al^3+^ is incorporated by Tschermaks substitution and only a small fraction is coupled to H^+^. Therefore, it is more likely that with increasing pressure water incorporation is more and more charge compensated by metal vacancies and less strongly promoted by Al^3+^, in agreement with Mierdel et al. (2007). This interpretation is further strengthened by the positive correlation between pressure and the absorbance of the band at 3687 and 3362 cm^−1^ (Fig. 2b). The latter has been assigned to Mg^2+^=2H^+^ (Prechtel and Stalder 2011), but concerning the 3687 cm^−1^ band, a consensus in recent literature has not been reached. On the one hand, the 3687 cm^−1^ band correlates with the silica content of the system and exhibits polarisation properties and a pressure trend similar to the band at 3593 cm^−1^, which has been assigned as hydrogarnet substitution (Si^4+^=4H^+^) by Prechtel and Stalder (2010, 2011). On the other hand, this band has been interpreted as a result of inclusions of amphibole (Skogby and Rossman 1989; Mosenfelder and Rossman 2013) that undoubtedly occur in natural samples. Since the stability of amphibole under the experimental conditions of this study can be ruled, we do not favour an interpretation of the band at 3687 cm^−1^ as a result of amphibole inclusions. However, other defect species such as planar defects are not excluded.

**Fig. 7:**
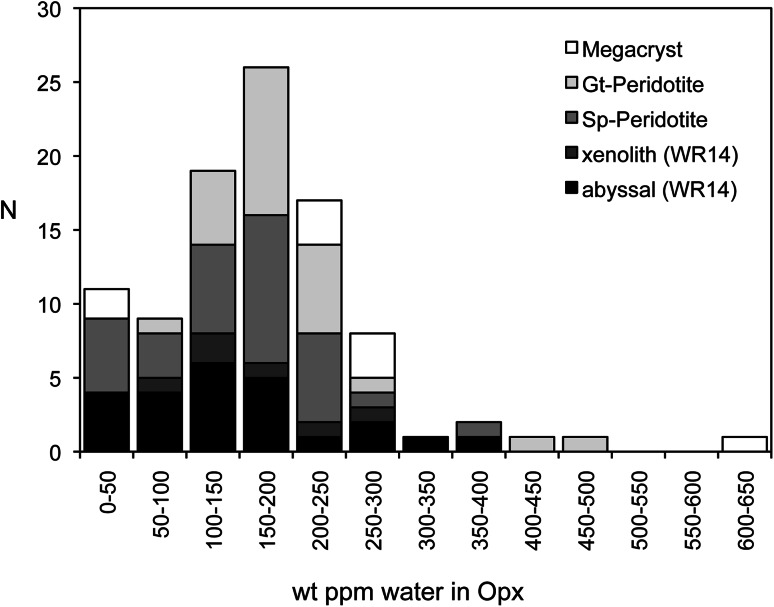
Compilation of published water contents in orthopyroxene (Bell and Rossman 1992; Bell et al. 2004; Grant et al. 2007; Peslier et al. 2002; Rossman 1996; Sundvall and Stalder 2011; Peslier et al. 2012; Mosenfelder and Rossman 2013; Warren and Hauri 2014) from different lithologies in the Earth’s upper mantle. Water contents for WH14 (Warren and Hauri 2014) were measured by SIMS, and all other samples were analysed by FTIR using the calibration of Bell et al. (1995)

Orthopyroxenes from upper mantle peridotite xenoliths typically show water concentrations that are only approximately one-tenth of those encountered in enstatite from the water-saturated experiments of this study (Fig. 7). The most important reason is that the water activity in the upper mantle is much lower than in the high-pressure experiments. The relatively low water content in mantle pyroxenes in general, as well as the high variability in water contents amongst mantle pyroxenes, cannot be explained by the trace metal chemistry in pyroxene (formation of anhydrous defects, such as Al–Cr-Tschermaks component, by mutual compensation of different Al- and Cr-induced OH-defects, e.g. Prechtel and Stalder 2012), because data points for Al-saturated enstatite from this study fall on the same trend line as that obtained by Mierdel et al. (2007) in the system MgO–Al_2_O_3_–SiO_2_–H_2_O (Fig. 5), illustrating the predominant importance of Al for the generation of OH-defects in enstatite. Fe^3+^, another metal impurity not encountered in our experiments, is expected to behave similar to Cr^3+^ and promotes coupled substitution involving protons during water-saturated crystallisation (Stalder 2004), and therefore is not judged relevant in this context either. Therefore, the variability in the water content of mantle orthopyroxenes most likely is due to variations in the water activity in the respective environment, triggered by processes such as melt depletion or metasomatism (Peslier et al. 2012; Warren and Hauri 2014), or by partial hydrogen loss as a result of oxidation of ferrous to ferric iron during uplift, leading in some cases to lower water contents than representative for upper mantle *P*–*T*–*f*O_2_ conditions (Skogby and Rossman 1989).

### Band ratios of OH absorptions in enstatite: relevance for natural systems

**Fig. 8:**
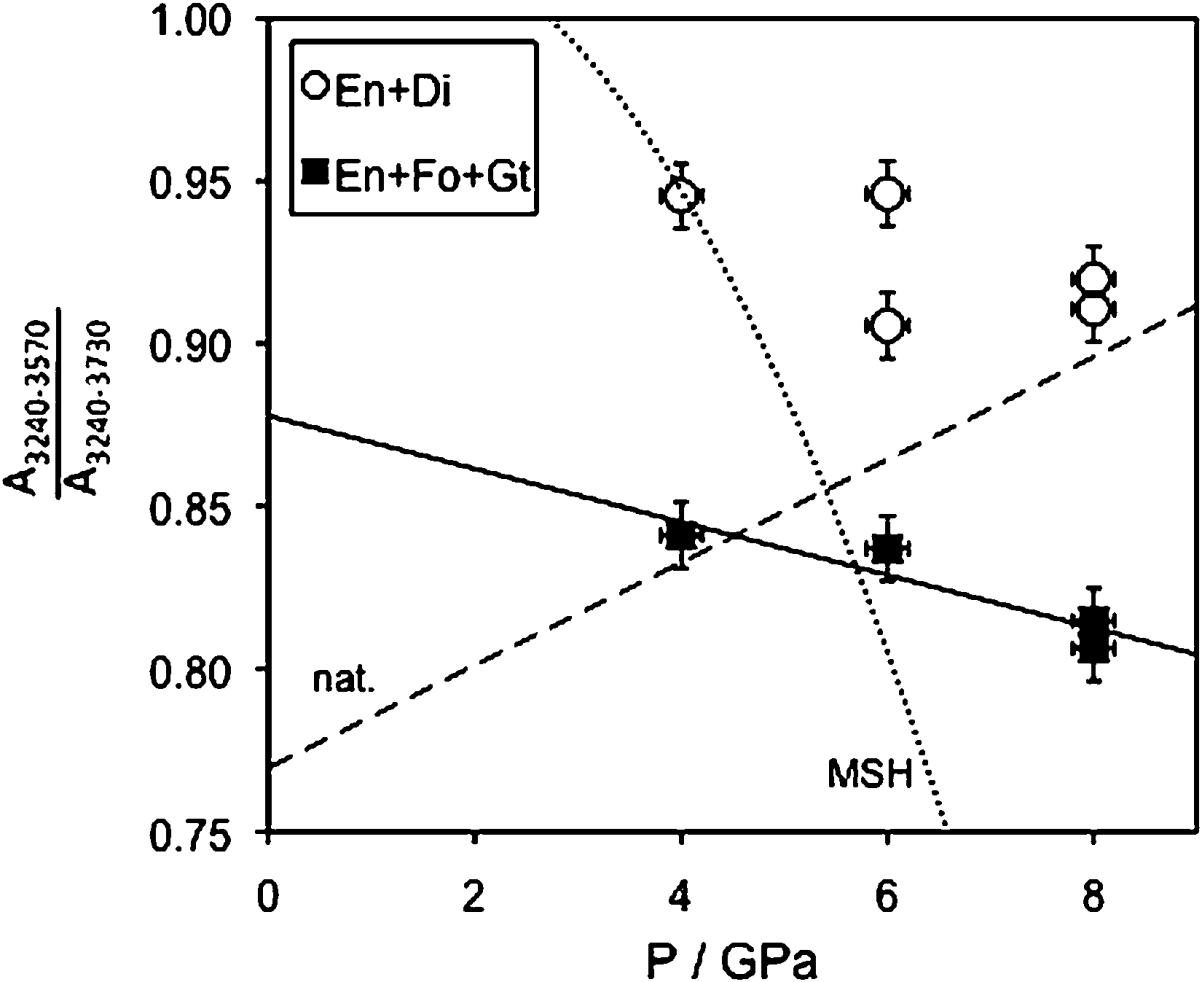
Correlation between band ratio *A*_3240–3570_/*A*_3240–3730_ and pressure for enstatite in phase assemblages En + Di and En + Fo + Gt. Trends for En + Fo in the pure MSH-system (Prechtel and Stalder 2011) and for natural mantle samples (Prechtel and Stalder 2012) are shown as *dotted* and *broken line*, respectively

One of the principal aims of this study is the identification of potentially pressure sensitive features of the IR-spectra of enstatite in the spectral region of OH-stretching vibrations. For an easier comparison with data from previous studies (e.g. Prechtel and Stalder 2012), a distinction between high- and low-wavenumber bands was made and the integral absorbance ratio (*A*_3240–3570_/*A*_3240–3730_) used as proxy for pressure. Results from this study are treated as two subsets based on the phase assemblages En + Di and En + Fo + Gt, respectively. Enstatite from both phase assemblages shows a similar and weak negative correlation between absorbance ratio (*A*_3240–3570_/*A*_3240–3730_) and pressure (Fig. 8), albeit with a shift of the trend for En + Di + Gt to significantly lower values. It has also to be noted that the observed pressure trend of (*A*_3240–3570_/*A*_3240–3730_) in natural samples (Prechtel and Stalder 2012) is mainly due to the different behaviour of the bands between 3600 and 3515 cm^−1^ and not due to the bands above 3600 cm^−1^ as in the present study (Fig. 2b).

Another pressure sensitive feature of the IR-spectra of enstatite in the assemblage En + Fo + Gt is the continuous shift of the *E*||*nγ* component of the OH-band generated by octahedral OH-defects. This band is observed at 3362 cm^−1^ at low levels of bulk Al (Fig. 2a) and in systems without any impurity cations (Prechtel and Stalder 2011). In case of Al-saturation at 4 GPa, it has a maximum at 3400 cm^−1^, which is shifted back to 3362 cm^−1^ at 8 GPa (Fig. 1c, 2b). As discussed above, this shift is probably caused by a reduced coupled incorporation of Al^3+^ and H^+^ and a concomitant increased amount of Mg^2+^=2H^+^ exchange, making the OH-defects in the octahedral site more similar to the OH-defect in the pure endmember. This pressure-dependent shift, however, is not observed in natural samples (cf. Prechtel and Stalder 2012) and therefore cannot be used as geobarometer.

If the absorption band at 3687 cm^−1^ in both investigated phase assemblages (Fig. 2b, c) is assigned to protonated Si-vacancies (Prechtel and Stalder 2010, 2011), its positive correlation with pressure may reflect a decreasing silica activity with increasing pressure. This phenomenon has been observed in partial melts from peridotitic systems that show decreasing silica contents with increasing pressure (Takahashi 1986). This effect is more pronounced in Al-saturated enstatite coexisting with garnet than in Al-undersaturated systems because with increasing pressure less tetrahedral Al is incorporated into enstatite. It should be noted that the saturation of an Al-rich phase (rather than coexistence with garnet) is the important factor, so the presence of spinel or plagioclase at lower-pressure regimes would also constrain the Al-specific OH-defects in enstatite.

**Fig. 9:**
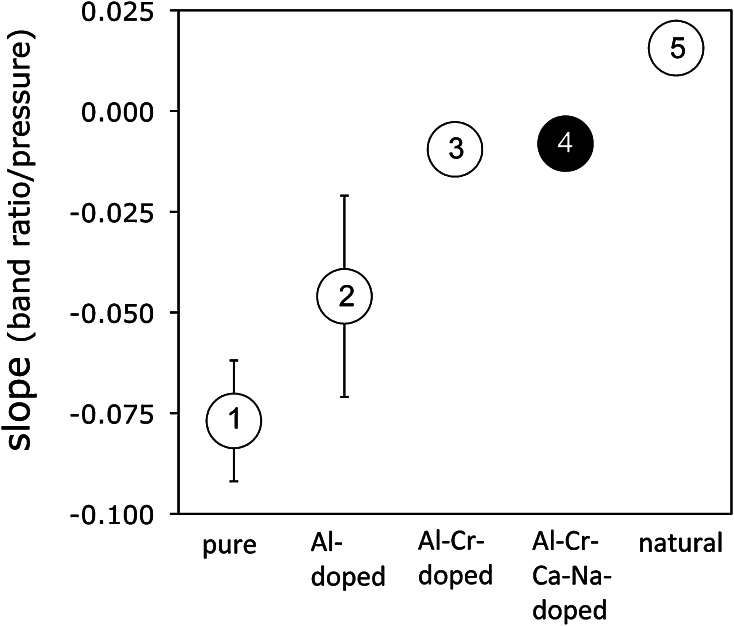
Evolution of the slope in the band ratio—pressure trend (Fig. 3) with increasing complexity of the chemical system. The large errors for *1* and *2* are due to the large range of band ratios. Data sources: *1* Prechtel and Stalder (2011), *2* Stalder (2004), *3* Prechtel and Stalder (2012), *4* this study, *5* Prechtel and Stalder (2012)

Based on the results of the present study, it is not possible to satisfactorily explain the discrepancy in H_2_O contents of enstatite between the synthetic bulk systems of this study and natural systems. One general problem for natural systems is broad and overlapping absorption bands that makes the quantitative characterisation of the defect chemistry and thermodynamic modelling very complicated. Although the observed trend of IR band ratios for enstatite from the Na–Ca–Al–Cr-doped system does not reproduce the trend observed in natural orthopyroxene from mantle xenoliths, the results of this study are a further step towards closing the gap between pure end member and natural system behaviour (Fig. 9), suggesting that it is justified to apply the band ratio (*A*_3240–3570_/*A*_3240–3730_) in natural enstatite as geobarometer.
